# Supplementary material for: Paternal multigenerational exposure to an obesogenic diet drives epigenetic predisposition to metabolic diseases in mice
Source: eLife. 2021 Mar 30;10:e61736. doi: 10.7554/eLife.61736 (PMC8051948; doi:10.7554/eLife.61736)
Supplement: Figure 1—source data 1. [file elife-61736-fig1-data1.docx]

**Figure 1-source data 1. Physiological characteristics of different WD groups**

| **Characteristic** | **Control**  **n=17** | **WD1**  **n=15** | **WD2**  **n=15** | **WD3**  **n=16** | **WD4**  **n=42** | **WD5**  **n=53** |
| --- | --- | --- | --- | --- | --- | --- |
| Body weight (g) (12 weeks) | 25.6(25-27) | 26(24.9-27.3) | 26.2(25.6-28.5) | 26.9(25.7-27.6) | **28.3(26.9-29.4) ***^1^** | **27.5(26.2-28.9)*** ^1^** |
| Body weight (g) (16 weeks) | 27.4(26.9-28.2) | **28.5(28-30)*** | **30(27.1-32)*** | 28.4(27.2-29.4) | **31.1(29.4-32.6)***^1^** | **30.4(28.9-32.6)***^1^** |
| Daily diet consumption (Kcal/day) | 10.2(10.2-10.3) | **11.3 (10.9-12.1)***** | **11.3(10.5-12.9)***** | **12.1(11.3-12.1)***** | **13.3(12.5-13.3)***** | **10.9(10.5-12.15)***** |
| Kidney (g) | 0.37(0.35-0.4) | 0.4(0.35-0.47) | 0.39(0.37-0.42) | 0.4(0.38-0.44) | **0.42(0.38-0.45)**** | **0.4(0.38-0.45)*** |
| Kidney/body weight (%) | 1.27(1.16-1.41) | 1.4(1.24-1.62) | 1.33(1.27-1.46) | 1.38(1.3-1.51) | 1.34(1.16-1.44) | **1.34(1.21-1.59)*** |
| Liver (g) | 1.4(1.26-1.5) | 1.53(1.3-1.65) | 1.56(1.4-1.7) | 1.45(1.28-1.63) | **1.83(1.67-2.01)***^1^** | **1.6(1.45-1.82)**** |
| Liver/body weight (%) | 5.1(4.8-5.3) | 5.4(4.8-5.6) | 5.2(5.0-5.8) | 5.3(4.9-5.8) | **5.7(5.4-6.3)***^1^** | **5.6(5.2-6)**** |
| gWAT (g) | 0.38(0.31-0.49) | **0.90(0.66-1.28)** | **0.94(0.82-1.15)***** | **1.1(0.75-1.24)***** | **1.25(0.8-1.7)***** | **1.24(1.01-1.56)***^1^** |
| gWAT/body weight (%) | 1.2(1.1-1.4) | **3.2(2.5-4.2)**** | **3.5(2.9-3.7)***** | **3.7(2.6-4)***** | **4.2(2.7-4.9)***** | **4.2(3.5-4.8)***** |
| Abdominal Adipose Volume (arbitrary unit) | 14(10-20)  (n=10) | 25(22-32)  (n=5) | 25(23-27)  (n=4**)** | **36(27-49)***  **(n=6)** | **33(26-49)****  **(n=7)** | **46(27-57)*****  **(n=11)** |

Values are expressed as median(IQR). Organ weight and abdominal adipose volume have been measured at the time of sacrifice of the mice (18 weeks). Numbers are in bold if p<0.05. * and 1 identified the WDs groups whose median rank difference was statistically significantly different as compared to that of the CD and WD1 groups, respectively. *p_adj_<0.05, **p_adj_<0.01, ***p_adj_<0.001.
