# Supplementary material for: Paternal multigenerational exposure to an obesogenic diet drives epigenetic predisposition to metabolic diseases in mice
Source: eLife. 2021 Mar 30;10:e61736. doi: 10.7554/eLife.61736 (PMC8051948; doi:10.7554/eLife.61736)
Supplement: Figure 3—source data 1. [file elife-61736-fig3-data1.docx]

|  |  |  |  |  |
| --- | --- | --- | --- | --- |

**Figure 3-source data 1. Physiological characteristics of F1, F2 and F3 male progenies from either WD1 or WD5 males**

| **Characteristic** | **Control**  **n=20** | **F1** | | | **F2** | | | **F3** | | |
| --- | --- | --- | --- | --- | --- | --- | --- | --- | --- | --- |
|  |  | **WD1**  **n=10** | **WD5**  **n=17** | | **WD1**  **n=14** | **WD5**  **n=17** | | **WD1**  **n=10** | **WD5**  **n=17** | |
| Body weight (g) (12 weeks) | 25.6(25-27) | **28.3 (27.2- 29.8)***** | | **29.3(27.3-29.9)**** | 27.1(26.1-28.2) | | **28.9(27.7-29.8)***^1^** | 25.6(25-25.8) | | **29.4(27.2-30.3)**^1^** |
| Body weight (g) (16 weeks) | 27.6(26.9-29.7) | **30(28.8-31.7)*** | | **29.1(28.3-30.6)*** | **30.3(29-31.5)**** | | **31.4(29.7-31.8)***** | 27.8(26.9-30.2) | | **30,7(28.7-33.2)***^1^** |
| Food intake (Kcal/d) | 10.3(10.2-10.4) | **11.6(11.3-11.8) **** | | 10.6(9.3-11.4) | 10.2(10.2-10.2) | | **11.7(10.3-12.3)***** | 10.2(10.2-10.3) | | 11(10-13.2) |
| Kidney (g) | 0.37(0.35-0.4) | 0.33(0.33-0.37) | | 0.39(0.36-0.42) | 0.39(0.34-0.43) | | 0.35(0.33-0.39) | 0.35(0.35-0.39) | | **0.45(0.41-0.52)*^1^** |
| Kidney/body weight (%) | 1.3(1.2-1.4) | 1.2(1.1-1.2) | | 1.3(1.2-1.4) | 1.4(1.2-1.5) | | 1.1(1.1-1.2) | 1.3(1.3-1.5) | | **1.2(1.1-1.4)*** |
| gWAT (g) | 0.38(0.31-0.49) | **0.49(0.43-0.52)*** | | **0.68(0.51-0.81)*** | **0.54(0.46-0.57)*** | | **0.66(0.47-0.75)***** | 0.38(0.29-0.39) | | **0.80(0.68-1)***^1^** |
| gWAT/body (%) | 1.2(1.1-1.4) | **1.6(1.74-1.7)*** | | **1.5(1.3-2.0)*** | **2.1(1.7-2.7)***** | | **2.1(1.5-2.4)***** | 1.2(1.0-1.4) | | **3.3(2.1-4.7)***^1^** |
| Liver (g) | 1.4(1.3-1.9) | 1.4(1.4-1.6) | | **1.5(1.3-1.8) *** | 1.(1.2-1.5) | | **1.6(1.5-1.7)*** | 1.4(1.3-1.6) | | **1.8(1.5-1.9)*** |
| Liver/body weight (%) | 5.1(4.8-5.3) | 4.9(4.6-5.1) | | 5.1(4.9-5.4) | **4.6(3.8-4.7)*** | | 5.1(4.8-5.3) | 5.0(4.8-5.2) | | 5.0(4.9-5.4) |
| Fasting Glucose (g) | 85(74-89) | 66(62-74) | | 78(67-87) | **66(64-73)*** | | **67(63-75)*** | 76(69-85) | | 73(67-95) |
| AUC-GTT (g/dl/min) | 18.9(16.5-20.8) | **24.8(21.9-26.5)*** | | **25.6(22.7-28.1)*** | **27.1(22.9-29.6)** | | 22.8(19.8-24.8) | 20.1(19.8-20.5) | | 21.6(18.2-24.9) |
| AUC-ITT (/dl/min) | 3.7(3.0-4.7) | 3.7(3.1-4.0) | | 3.2(2.8-4.3) | 4.0(3.6-4.5) | | 3.9(3.5-4.6) | 3.2(2.9-3.3) | | 3.9(3.5-4.7) |
| Total cholesterol (mg/dl) | 1.0(0.6-1.2) | 0.8(0.7-0.9) | | 1.0(0.8-1.3) | 0.7(0.5-0.8 | | 0.8(0.7-1.0) | nd | | nd |
| Abdominal Adipose Volume (mm^3^) | 1.4(1.1-2.1)  n=9 | **1.7(1.2-2.1)***  **n=6** | | **2.2(1.5-2.4)** n=7** | nd | | **2.7(1.7-3.1)****  **n=5** | nd | | nd |

Values are expressed as median(IQR). Numbers are in bold if p<0.05. * and 1 identified the WDs groups whose median rank difference was statistically significantly different as compared to that of the CD and WD1 groups, respectively. *p_adj_<0.05, **p_adj_<0.01, ***p_adj_<0.001, nd= not determined.
