# Supplementary material for: Paternal multigenerational exposure to an obesogenic diet drives epigenetic predisposition to metabolic diseases in mice
Source: eLife. 2021 Mar 30;10:e61736. doi: 10.7554/eLife.61736 (PMC8051948; doi:10.7554/eLife.61736)
Supplement: Figure 3—source data 2. [file elife-61736-fig3-data2.docx]

**Figure 3-source data 2. Physiological characteristics of F1, F2 and F3 female progenies from either WD1 or WD5 males**

| **Characteristic** | **Control**  **n=11** | **F1** | | **F2** | | | **F3** | | |
| --- | --- | --- | --- | --- | --- | --- | --- | --- | --- |
|  |  | **WD1**  **n=10** | **WD5**  **n=15** | **WD1**  **n=9** | **WD5**  **n=15** | | **WD1**  **n=7** | **WD5**  **n=13** | |
| Body weight (g) (12 weeks) | 20.5(19.1-20.9) | **21.5(21-22.8)*** | 20.8(20.3-21.7) | 20.6(19.5-21.4) | | **21.7(20.7-22.2)*^1^** | 20.7(19.6-20.9) | **22.8(22.1-23.7)***^1^** | |
| Body weight (g) (16 weeks) | 21.4(20.9-21.8) | **22.3(21.5-24.0)*** | 22(20.9-23.1) | 22.3(21.0-23.5) | | **23.8(21.8-24)**** | 21.4(21.0-21.8) | **23.8(22.4-25.5)**^1^** | |
| Food intake (Kcal/d) | 9(8-9.6) | 9.3(9-9.3) | 9.3(8.6-9.3) | 9.7(9.3-9.7) | | 9(8.8-9) | 8.5(8-9.1) | **9.2(9-9.6)***^1^** | |
| Kidney (g) | 0.27(0.25-0.27) | 0.27(0.24-0.29) | 0.3(0.26-0.32) | 0.29(0.27-0.31) | | 0.28(0.26-0.29) | 0.27(0.26-0.28) | 0.28(0.26-0.3) | |
| Kidney/body weigt (%) | 1.2(1.1-1.2) | 1.2(1.1-1.2) | 1.3(1.1-1.4) | 1.3(1.2-1.3) | | 1.1(1.0-1.2) | 1.1(1.0-1.2) | 1.2(1.1-1.4) |  |
| gWAT (g) | 0.27(0.24-0.32) | 0.36(0.26-0.45) | **0.4(0.28-0.75) *** | 0.24(0.17-0.37) | | **0.5(0.4-0.9)**^1^** | 0.24(0.23-0.27) | **0.45(0.4-0.6)***^1^** | |
| gWAT/body weight (%) | 1.1(1.0-1.2) | **1.6(1.2-1.9)*** | 1.7(1.0-2.1) | 1.3(1.1-1.5) | | **1.8(1.6-2.1)*** | 1.1(0.9-1.3) | **1.7(1.3-2.0)*^1^** | |
| Liver (g) | 1.1(1.0-1.2) | 1.1(1.0-1.1) | 0.9(0.7-1.1)* | 1.2(1.0-1.3) | | 1.1(1.1-1.2) | 1.1(1.0-1.1) | 1.1(1.1-1.3) | |
| Liver/body weight (%) | 5.1(4.9-5.4) | 4.5(4.4-5.0) | **3.6(3.3-4.5)*** | **4.6(3.8-4.6)*** | | 4.8(4.3-4.9) | 4.8(4.7-5.0) | 4.9(4.6-5.0) | |
| Fasting Glucose (g) | 65(59-70) | 70(69-80) | 72(56-78) | 62(57-66) | | 70(65-81) | 69(65-72) | 68(60-77) | |
| AUC-GTT (g/dl/min) | 14.8(13.7-17.8) | 19.7(16.8-21.5) | **21.9(19.7-24.3)*** | **28.4(22.3-29.2)***** | | 17.8(16.4-19.6) | 19.5(14.3-21.5) | 21.4(18.0-24.4) | |
| AUC-ITT (mg/dl/min) | 3.1(2.9-3.3) | 3.1(2.7-3.4) | 2.9(2.7-3.3) | **4.0(3.6-4.5)*** | | 3.0(2.7-3.4) | 3.1(2.9-3.3) | 4.0(2.6-4.4) | |
| Total Cholesterol (mg/dl) | 0.6(0.5-0.6) | 0.6(0.4-0.6) | 0.6(0.4-0.7) | nd | | 0.7(0.6-0.7) | nd | nd | |

Values are expressed as median(IQR). Numbers are in bold if p<0.05. Numbers are in bold if p<0.05. * and 1 identified the WDs groups whose mean rank difference was statistically significantly different as compared to that of the CD and WD1 groups, respectively. *p_adj_<0.05, **p_adj_<0.01, ***p_adj_<0.001, nd= not determined.
