# Supplementary material for: Paternal multigenerational exposure to an obesogenic diet drives epigenetic predisposition to metabolic diseases in mice
Source: eLife. 2021 Mar 30;10:e61736. doi: 10.7554/eLife.61736 (PMC8051948; doi:10.7554/eLife.61736)
Supplement: Figure 5—source data 1. [file elife-61736-fig5-data1.docx]

**Figure 5-source data 1. Physiological characteristics of F1 male and female progenies RNA microinjected embryos**

|  | **F1-RNA male progenies** | | | **F1-RNA female progenies** | | |
| --- | --- | --- | --- | --- | --- | --- |
| **Characteristic** | **RNA-CD**  **n=9** | **RNA-WD1**  **n=12** | **RNA-WD5**  **n=38** | **RNA-CD**  **n=10** | **RNA-HFD1**  **n=6** | **RNA-WD5**  **n=30** |
| Body weight (g) (12 weeks) | 30.3(29-31) | **31.3(29.8-32)*** | **31.3(30.2-33.1)*** | 23.5(22.7-24.2) | 22.9(21.9-24.3) | 23.4(22.0-25.0) |
| Body weight (g) (16 weeks) | 33.8(33.0-34.0) | 33.0(32.1-34.4) | 33.0(31.1-34.4) | 26.7(25.2-27.4) | 24.1(23.9-27.8) | 25.5(24.0-26.9) |
| Kidney (g) | 0.5(0.5-0.5) | 0.4(0.4-0.5) | 0.47(0.4-0.56) | 0.3(0.3-0.37) | 0.3(0.3-0.3) | 0.35(0.3-0.4) |
| Kidney to body mass ratio (%) | 1.3(1.3-1.4) | 1.2(1.0-1.4) | 1.4(1.0-1.7) | 1.0(0.9-1.3) | 1.1(1.0-1.2) | 1.2(1.1-1.3) |
| gWAT (g) | 0.9(0.5-1.4) | 0.8(0.6-1.0) | 1.0(0.6-1.5) | 0.8(0.4-0.9) | 0.6(0.4-0.8) | 0.6(0.5-1.1) |
| gWAT to body mass ratio (%) | 1.8(1.4-3.1) | 2.3(1.8-3.2) | 2.7(1.5-2.9) | 2.7(1.5-3.0) | 2.3(1.6-3.0) | 2.3(1.6-3.7) |
| Liver (g) | 1.6(1.2-1.9) | 1.7(1.3-1.9) | 1.86(1.5-1.9) | 1.4(1.2-1.6) | **1.1(1.1-1.2)**** | 1.4(1.2-1.5) |
| Liver to body mass ratio (%) | 4.1(3.5-5.0) | 4.8(4.2-5.1) | 4.6(4.2-5.1) | 4.8(4.3-5.1) | **4.1(3.7-4.5)*** | 4.7(4.3-5.1) |
| Fasting Glucose (mg/dl) | 101(90-120) | 103(97-111) | **111(102-124)*** | 112(103-122) | **122(101-106)*** | 101(87-106) |
| AUC-GTT (g/dl/min) | 36.7(32.3-40) | 35.1(33.3-44.5) | **36(32.9-40.2)*** | 31.3(27.6-35.7) | **30.3(26.5-31.7)*** | 27.5(26.4-39.1) |
| AUC-ITT (mg/dl/min) | 6468±976 | **8751±490*** | **8139±349*** | 7.2(7.0-7.5) | 6.7(6.5-7.9) | 8.1(7.0-8.9) |

Values are expressed as median(IQR). Numbers are in bold if p<0.05. * identified the WDs groups whose mean rank difference was statistically significantly different as compared to that of the CD. *p_adj_<0.05, ** p_adj_ <0.01, *** p_adj_ <0.001.

.
