# Supplementary material for: Paternal multigenerational exposure to an obesogenic diet drives epigenetic predisposition to metabolic diseases in mice
Source: eLife. 2021 Mar 30;10:e61736. doi: 10.7554/eLife.61736 (PMC8051948; doi:10.7554/eLife.61736)
Supplement: Figure 5—source data 2. [file elife-61736-fig5-data2.docx]

**Figure 5-source data 2. Physiological characteristics of F2 male and female progenies RNA microinjected embryos**

| **Characteristic** | **F2-RNA male progenies** | | | | **F2-RNA female progenies** | | |
| --- | --- | --- | --- | --- | --- | --- | --- |
|  | **RNA-CD**  **n=11** | | **RNA-WD1**  **n=11** | **RNA-WD5**  **n=24** | **RNA-CD**  **n=11** | **RNA-WD1**  **n=8** | **RNA-WD5**  **n=18** |
| Body weight (g) (12 weeks) | | 26.9(25.8-27.8) | **30.2(28.3-31) **** | **28.7(28.0-29.4)**** | 22.2(21.8-22.7) | **25.0(22.9-25.6) *** | 21.5(20.3-22.1) |
| Body weight (g) (16 weeks) | | 29.0(28.1-30) | **32.0(28.5-35.5)*** | **31.0(30.2-32) **** | 22.4(21.4-23-0) | **26.9(23.3-27.6) *** | 22.7(21.2-23.7) |
| Kidney (g) | | 0.38(0.36-0.4) | 0.37(0.3-0.4) | 0.4(0.35-0.47) | 0.3(0.3-0.7) | 0.3(0.28-0.33) | 0.3(0.28-0.36) |
| Kidney to body mass ratio (%) | | 1.2(1.1-1.3) | 1.2(1.0-1.4) | 1.5(1.0-1.7) | 1.1(1.0-1.3) | 1.2(1.0-1.2) | 1.1(0.9-1.3) |
| gWAT (g) | | 0.6(0.3-0.6) | **1.0(0.5-1.4) *** | 0.7(0.5-0.9) | 0.5(0.3-0.6) | **1.0(0.5-1.2)*** | **0.6(0.5-1.2)*** |
| gWAT to body mass ratio (%) | | 2.2(1.5-2.6) | 3.3(1.7-4.1) | 2.3(2.1-3.4) | 1.7(0.9-2.1) | **2.2(1.3-3.3)** | **2.9(2.2-3.7)*** |
| Liver (g) | | 1.4(1.2-1.6) | **1.8(1.6-1.9)**** | 1.6(1.4-1.7) | 1.2(1.1-1.5) | 1.2(1.1-1.5) | 1.2(1.1-1.4) |
| Liver to body mass ratio (%) | | 4.8(4.3-5.1) | 5.1(4.9-5.4) | 4.8(4.5-5.3) | 4.1(3.6-4.5) | 4.5.0(4.6-5.5) | 4.6(3.9-4.9) |
| Fasting Glucose (mg/dl) | | 183(168-200) | 207(194-215) | 163(125-176) | 153(145-160) | 140(117-184) | 160(157-171) |
| AUC-GTT (mg/dl/min) | | 30.3(30.3-34.2) | 34.5(30.4-37.8) | 31.3(29.5-36.5) | 29.7(28.3-30.1) | 31.6(28.3-33.9) | 30(27.8-34.3) |
| AUC-ITT (mg/dl/min) | | 7.3(6.5-9.3) | **12.4-9.7-14.5)*** | 6.8(4.8-11.3) | 5.1(4.7-7.7) | **6.8(6.2-7.5)*** | **6.5(5.5-7.8)*** |

Values are expressed as median(IQR). Numbers are in bold if p<0.05. * identified the WDs groups whose mean rank difference was statistically significantly different as compared to that of the CD. *p_adj_<0.05, ** p_adj_ <0.01, *** p_adj_ <0.001.
