# Supplementary material for: Paternal multigenerational exposure to an obesogenic diet drives epigenetic predisposition to metabolic diseases in mice
Source: eLife. 2021 Mar 30;10:e61736. doi: 10.7554/eLife.61736 (PMC8051948; doi:10.7554/eLife.61736)
Supplement: Figure 5—source data 3. [file elife-61736-fig5-data3.docx]

**Figure 5-source data 3. Physiological characteristics of F3 male and female progenies RNA microinjected embryos**

| **Characteristic** | | **F3-RNA male progenies** | | | **F3-RNA female progenies** | | | |
| --- | --- | --- | --- | --- | --- | --- | --- | --- |
|  |  | **RNA-CD**  **n=6** | **RNA-WD1**  **n=8** | **RNA-WD5**  **n=15** | **RNA-CD**  **n=12** | **RNA-WD1**  **n=10** | **RNA-WD5**  **n=13** | |
| Body weight (g) (12 weeks) | 26.5(26.0-27.8) | | 27.6(27.0-29.0) | 28.1(26.5-29) | 21.0(20.3-22.1) | 20.2(19.6-22.5) | | 20.9(20.1-22.1) |
| Body weight (g) (16 weeks) | 29.0(28.1-30.0) | | 29.3(28.8-30.9) | 30.0(29.0-30.8) | 21.8(21.2-22.7) | 22.3(20.9-23.5) | | 22.4(21.4-24) |
| Kidney (g) | 0.38(0.36-0.4) | | 0.45(0.37-0.54) | 0.38(0.36-0.4) | 0.3(0.3-0.3) | 0.3(0.3-0.3) | | 0.3(0.3-0.37) |
| Kidney (%) | 1.2(1.0-1.3) | | 1.2(1.0-1.4) | 1.1(1.0-1.2) | 1.2(1.0-1.2) | 1.1(1.0-1.2) | | 1.1(1.0-1.5) |
| gWAT (g) | 0.6(0.3-0.7) | | 0.8(0.6-0.8) | 0.9(0.7-1.0) | 0.5(0.4-0.5) | 0.3(0.2-0.5) | | 0.4(0.3-0.6) |
| gWAT (%) | 2.5(1.9-2.6) | | 2.6(2.2-2.7) | 2.8(2.3-3.0) | 1.6(1.1-1.8) | 1.2(1.1-1.9) | | 1.5(1.3-2.5) |
| Liver (g) | 1.4(1.2-1.4) | | **1.8(1.5-1.8)*** | **1.6(1.4-1.8)*** | 1.2(0.9-1.3) | 1.2(1.1-1.3) | | 1.3(1.0-1.4) |
| Liver (%) | 4.4(4.2-4.5) | | 4.9(4.0-5.0) | 4.4(4.1-4.6) | 4.2(4.1-4.7) | 4.8(4.3-4.9) | | 5.2(3.8-5.3) |
| Fasting Glucose (mg/dl) | 161(101-191) | | 162(109-186) | **214(175-232)*** | 153(145-160) | 185(168-192) | | 173(151-182) |
| AUC-GTT (g/dl/min) | 30.6(30.2-35) | | 34(27-38.4) | **45.7(30.7-48.9)**** | 29.7(28.3-30) | 31.6(28.3-33.9) | | 30.0(27.8-34.3) |
| AUC-ITT (g/dl/min) | 7.7(6.8-10.9) | | 7.9(7.0-10.8) | **13.5(13.1-14.4)*** | 7.9(6.9-8.7) | **8.4(6.2-10.2)**** | | **10.1(7.2-12.2)**** |

Values are expressed as median(IQR). Numbers are in bold if p<0.05. * identified the WDs groups whose mean rank difference was statistically significantly different as compared to that of the CD. *p_adj_<0.05, ** p_adj_ <0.01, *** p_adj_ <0.001, nd= not determined.
