# Supplementary material for: Paternal multigenerational exposure to an obesogenic diet drives epigenetic predisposition to metabolic diseases in mice
Source: eLife. 2021 Mar 30;10:e61736. doi: 10.7554/eLife.61736 (PMC8051948; doi:10.7554/eLife.61736)
Supplement: Figure 5—source data 4. [file elife-61736-fig5-data4.docx]

**Figure 5-source data 4. Physiological characteristics of F4 male and female progenies RNA microinjected embryos**

| **Characteristic** | **F4-RNA male progenies** | | | **F4-RNA female progenies** | | |
| --- | --- | --- | --- | --- | --- | --- |
|  | **RNA-CD**  **n=6** | **RNA-WD1**  **n=8** | **RNA-WD5**  **n=10** | **RNA-CD**  **n=6** | **RNA-WD1**  **n=6** | **RNA-WD5**  **n=9** |
| Body weight (g) (12 weeks) | 28.8(27.7-29.4) | 26.1(23.7-28.7) | 28.5(27.9-29.4) | 21.720.9-22.8) | 20.0(18.8-21.4) | 21.3(20.6-21.8) |
| Body weight (g) (16 weeks) | 29.8(29.4-30.4) | 28.3(26.9-30.6) | 29.0(27.8-30.8) | 21.4(20.9-22.8 | 21.3(19.9-22.1) | 21.8(21.2-23.0) |
| Fasting Glucose (mg/dl) | 175(156-207) | 145(131-163) | 164(146-250) | 152(143-162) | 178(160-197) | 164(156-186) |
| AUC GTT(mg/dl) | 31.8(29.1-35) | 34.4(31.0-36.7) | 34.0(30.8-39.6) | 31.3(27.7-35.7) | 30.3(26.5-31.8) | 27.5(26.4-39.0) |
| AUC ITT (mg/dl) | 7.6(6.8-10.9) | 7.9(7.1-10.8) | 13.5(13.1-14.4) | 7.8(6.5-9.8) | 7.5(7.1-9.0) | 7.9(6.1-8.8) |

Values are expressed as median(IQR). Numbers are in bold if p<0.05. * identified the WDs groups whose mean rank difference was statistically significantly different as compared to that of the CD. *p_adj_<0.05, ** p_adj_ <0.01, *** p_adj_ <0.001.
