# Supplementary material for: Paternal multigenerational exposure to an obesogenic diet drives epigenetic predisposition to metabolic diseases in mice
Source: eLife. 2021 Mar 30;10:e61736. doi: 10.7554/eLife.61736 (PMC8051948; doi:10.7554/eLife.61736)
Supplement: Transparent reporting form [file elife-61736-transrepform.pdf]

eLIFE
1st Floor
24 Hills Road
Cambridge CB2 1JP, UK
P 01223 855340
W elifesciences.org
T @elife

eLife's transparent reporting form

We encourage authors to provide detailed information *within their submission* to facilitate the interpretation and replication of experiments. Authors can upload supporting documentation to indicate the use of appropriate reporting guidelines for health-related research (see EQUATOR Network), life science research (see the BioSharing Information Resource), or the ARRIVE guidelines for reporting work involving animal research. Where applicable, authors should refer to any relevant reporting standards documents in this form.

If you have any questions, please consult our Journal Policies and/or contact us: editorial@elifesciences.org.

Sample-size estimation

- You should state whether an appropriate sample size was computed when the study was being designed
- You should state the statistical method of sample size computation and any required assumptions
- If no explicit power analysis was used, you should describe how you decided what sample (replicate) size (number) to use

Please outline where this information can be found within the submission (e.g., sections or figure legends), or explain why this information doesn't apply to your submission:

According to our previous publication (Grandjean V, Fourre S, De Abreu DA, Derieppe MA, Remy JJ, Rassoulzadegan M. RNA-mediated paternal heredity of diet-induced obesity and metabolic disorders. Sci Rep. 2015;5:18193), we decided to use at least 10 mice/group in our study. This sample-size has proven to be sufficient to observe significant differences (pvalue<0.05) in several physiological parameters. On the other hand, to avoid experimental bias, the experimental procedures were repeated twice (Figure legends (≠1 and 3) and Material and methods).

Replicates

- You should report how often each experiment was performed
- You should include a definition of biological versus technical replication
- The data obtained should be provided and sufficient information should be provided to indicate the number of independent biological and/or technical replicates
- If you encountered any outliers, you should describe how these were handled
- Criteria for exclusion/inclusion of data should be clearly stated

High-throughput sequence data should be uploaded before submission, with a private link for reviewers provided (these are available from both GEO and

- ArrayExpress). Please outline where this information can be found within the submission (e.g., sections or figure legends), or explain why this information doesn't apply to your submission:

To avoid experimental bias, the experimental procedures were repeated twice (Figure legends ≠1 and 3).<br>For us, biological replicate corresponds to distinct mouse. Each sample-size is indicated in the corresponding figure legend.<br>High-throughput sequence have been deposited in the GEO Database (Material and Methods)

Statistical reporting

- Statistical analysis methods should be described and justified
- Raw data should be presented in figures whenever informative to do so (typically when N per group is less than 10)
- For each experiment, you should identify the statistical tests used, exact values of N, definitions of center, methods of multiple test correction, and dispersion and precision measures (e.g., mean, median, SD, SEM, confidence intervals; and, for the major substantive results, a measure of effect size (e.g., Pearson's r, Cohen's d)
- Report exact p-values wherever possible alongside the summary statistics and 95% confidence intervals. These should be reported for all key questions and not only when the p-value is less than 0.05.

Please outline where this information can be found within the submission (e.g., sections or figure legends), or explain why this information doesn't apply to your submission:

Statistical analyses were performed using the Kruskal-Wallis test followed by the two-stage step-up method of Benjamini, Krieger and Yekuteil for multiple comparisons of body weight, body composition, cholesterol, and leptin levels, as well as leptin mRNA expression and AUC-GTT and AUC-ITT between the WD cohorts, F1-, F2-, and F3-progenies and RNA-microinjected progenies.<br>To measure the linear relationship between two variables, we used Spearman's correlation coefficient. All statistical analyses were performed with Prism 7 for Mac OS X software (GraphPad software, Inc.). Data are presented as the median ± SD. A *p* value of <0.05 was considered statistically significant.<br>Sample size and replicates are indicated in the figure legends. The WD cohort and WD progenies were repeated twice. (Described in material and Methods).<br>This information can be found in Material and Methods and in Figure Legends.

Group allocation

- Indicate how samples were allocated into experimental groups (in the case of clinical studies, please specify allocation to treatment method); if randomization was used, please also state if restricted randomization was applied
- Indicate if masking was used during group allocation, data collection and/or data analysis

Please outline where this information can be found within the submission (e.g., sections or figure legends), or explain why this information doesn't apply to your submission:

No masking was used during group allocation. However, masking was used during data analysis.

Additional data files ("source data")

- We encourage you to upload relevant additional data files, such as numerical data that are represented as a graph in a figure, or as a summary table
- Where provided, these should be in the most useful format, and they can be uploaded as "Source data" files linked to a main figure or table
- Include model definition files including the full list of parameters used
- Include code used for data analysis (e.g., R, MatLab)
- Avoid stating that data files are "available upon request"

Please indicate the figures or tables for which source data files have been provided:

Supplementaries tables 1-10 and GEO Database with accession number GSE148972 and GSE138989.
